# Supplementary material for: Inflammatory and immunopathological differences in brains of permissive and non-permissive hosts with Angiostrongylus cantonensis infection can be identified using 18F/FDG/PET-imaging
Source: PLoS Negl Trop Dis. 2024 May 28;18(5):e0012188. doi: 10.1371/journal.pntd.0012188 (PMC11161054; doi:10.1371/journal.pntd.0012188)
Supplement: Attachment — Submitted filename: Response letter 240329.doc [file pntd.0012188.s004.doc]

Dear Editor: Apr. 1 2024

Attached, please find our revised manuscript entitled “**Inflammatory and immunopathological differences in brains of permissive and non-permissive hosts with *Angiostrongylus cantonensis* infection can be identified using ^18^F/FDG/PET-imaging”**. The previous reference number of this paper is No. **PNTD-D-23-01499.**

We greatly appreciate all the valuable comments that have offered. These comments have helped us in improving the quality of the manuscript. The revisions are listed below for review, the changes we have made are highlighted with **BOLD** word in the text and their locations in the manuscript are indicated in parentheses. Please review the updated manuscript and kindly let us know if additional changes are necessary. In addition, we also added a new author: **Tzu-Yuan Lin** who helps us for finishing in revision. We sincerely hope that the revised manuscript and this letter will furnish sufficient explanation for the reviewer’s questions.

Sincerely yours,

Po-Ching Cheng, Ph.D.

Department of Molecular Parasitology and Tropical Diseases,

School of Medicine, College of Medicine,

Taipei Medical University,

Taipei, Taiwan

Reviewers' Comments:

**Reviewer #1:**

1. The author mentioned in the text that ICR mice and SD rats were infected respectively with 50 and 100 L3. How is the number of infected larvae decided, and how the definite infected numbers are sure?

**Ans: Thank you for your question. The number of infected larvae follows our previous studies and has been added in the Materials section as references 38 and 39. When infecting animals, we use a microscope to pick out L3 larvae, allowing us to calculate the number of larvae required to infect an animal. After infection, we flushed the feeding tubes and check for remaining larvae to determine the precise number of infections. (Page8 line 145-146, ref. 38, 39)**

2. The FDG PET mouse results in Figure 1A reveal that there are obvious signals in the cerebellum and brainstem at the second week, but the data in Figure 1C only shows a value increase in the cerebellum. Why are there such differences between the cerebellum and brainstem? Please explain clearly how to distinguish them.

**Ans:** **Thank you for the comments. We have selected representative images of PET result to present in Figure 1A, while Figure 1C shows the statistical analysis results of all data. Although there were obvious signals in the cerebellum and brain stem of representative image, all values combined showed significant differences only in the cerebellum of the second week of infection. Our analysis uses the mouse and rat brain partition modules in the software to depict real-time PET brain values and obtain radiometric quantification after the system performs a selection. The average FDG accumulation was higher in the cerebellar locations of the all mice, while not all mice showed high accumulation in the brainstem. Therefore, although there was signal in the brainstem image in Figure 1A during the second week, the data showed that the increase was not as significant as in the cerebellum.** **We have added relevant descriptions to the text. (Page17 line 313)**

3. Fig 3A show Iba-1 in IHC gradually increase with the time of infection and peak at the 4th week; however, the expression of Iba-1 gene and protein in qPCR and WB increase significantly in the second week than the 4th week? Why they show different?

**Ans:** **Thank you for your comment. In IHC experiments, the area where we observed and quantified Iba-1 expression changes mainly focused around the limbic system of the brain, while gene and protein experiments were extracted from the entire brain region, so the trends between the two may be different. We concluded that the significant increase in Iba-1 expression throughout the brain region during the second week of infection was due to penetration of parasite antigens into limbic system of brains, activating microglia and subsequent activation of YM-1 eosinophil responses. Most microglia are distributed around the blood vessels of the limbic system of the brain. In comparison, they are evenly distributed in the parenchymal area of cerebral cortex. Therefore, the Iba-1 expression trend may be diluted in whole brain, while the periphery of the limbic system still shows a gradually and continuous increase trend of expression in infection. Previous studies have also reported that when the blood-brain barrier (BBB) is disrupted, Iba-1 will respond preferentially, activate and migrate to the injured site. Since *A. cantonensis* will easily break through the blood-brain barrier and enter the interior of the brain such as the limbic system and cerebellum in non-permissive host's brains, Iba-1 in IHC gradually increase with the time of infection, whereas the genes and the proteins increased significantly only in the second week without the 3rd-4th week.** **Relevant references are listed below and added to the Discussion section in text. (Page30-31 line 516-525, ref. 52, 53, 54)** **[****1-4****]**

4. In the ELISA experiment, only mice sera were detected for IL5 without rats? please explain.

**Ans: Thank you for your comment.** **Previous studies have shown that IL-5 participates in the inflammatory response by activating the TH2 response and amplifying eosinophil activation. However, we did not observe very severe inflammation within the brain of rats infected with *A. cantonensis*. Our previous study has also shown that eosinophils in the blood of mice infected with *A cantonensis* will increase to varying degrees. Therefore, here we focus on detecting IL-5 in mouse serum to observe whether it is related to inducing symptoms of severe eosinophilic meningitis, and compare it with the trend of PET values in the brain. Relevant references are listed below and added to the text. (Page32 line 559, ref. 38, 61, 62; Fig.5B)[****5-7****]**

**Reviewer #2:**

1. In the Methodology section, ensure consistency by describing actions in the past tense, as opposed to the predominantly future tense used by the authors. Please revise accordingly.

**Ans: We thank the comment of reviewer and apologize for any mistakes that have appeared in the article. We have reviewed our English grammar and correct all the mistakes accordingly in the manuscript. (Throughout the text)**

2. In the text and Table 1 (p.15), the authors exclusively described the worm burden in the brain of ICR mice. For enhanced clarity and understanding, it would be beneficial to explicitly mention, for example, that worms were not detected in the hearts of ICR mice. (Note: The authors touch upon this point in the Discussion section)

**Ans: Thank you for the comments. We have revised the description of results to explicitly mention that worms were not detected in the hearts of ICR mice for all. (Page15 line 270)**

3. Considering that eosinophils play a pivotal role as key effector inflammatory cells in cerebral Angiostrongyliasis, the authors should quantitatively assess the extent of eosinophil infiltration in different brain regions in correlation with YM-1 expression, rather than relying on descriptive wording. Similarly, a quantitative or semi-quantitative grading (i.e., 0, +, ++, +++) should be applied to assess microglia. If a direct count is challenging, a semi-quantitative approach would be acceptable.

**Ans: Thank you for your comment. We have supplemented Fig.2B with eosinophil counting to elucidate the trends in eosinophils associated with YM-1 expression between rats and mice during infection. We have also modified the relevant descriptions in the Results section. Additionally, microglia were quantified by Iba-1 IHC staining and software counting, as shown in Figure 3A. Iba-1 has been reported as a microglia-specific marker and is widely used in microglia detection, which can be used as microglia assessing quantitatively. The relevant data have been shown in Fig.3A-3 and descripted in the results. (page 11 line 195-197; page 18 line 323, 325-328; page 20 line 352-355; Fig.2A, 2B; page18-19 line 335-340; Fig.3A3)**

4. To complement the aforementioned suggestion, in Fig. 2, the histopathology of eosinophil and microglia infiltration should be highlighted either as insets or as separate panels. For example, Fig. 2A could represent the current figures, while Fig. 2B could focus specifically on eosinophil and microglia infiltration with high magnification.

**Ans: We are appreciated the comment. We have supplemented Fig. 2A2 to focus specifically on eosinophil and microglia infiltration with high magnification as the reviewer's suggestion. We also revised the related description in Figure legend. (page 18 line 325-328; page 20 line 354; Fig.2A2)**

5. The authors present a comprehensive study on the use of 18F-FDG PET imaging to diagnose Angiostrongylus cantonensis infection in the brains of both permissive (rats) and non-permissive (mice) hosts, examining inflammatory responses over a 4-week period. Their findings reveal significant 18F-FDG uptakes in the cerebellum, brainstem, and limbic system of mice, contrasting with the absence of such uptake in rat brains. Immunohistochemical staining and western blotting demonstrate a substantial increase in macrophage-derived microglia (Iba-1 positive) and the eosinophil chemotactic factor, YM-1, in mice brains, with a less pronounced effect observed in rats. Serum levels of systemic proinflammatory cytokines (TNF, IFN-γ, IL-2) and anti-inflammatory cytokines (Th2, IL4, IL-5) differ between mice and rats, though conclusions are not yet definitive. The authors propose that 18F-FDG uptake in the host brain may be attributed to the accumulation of immune cells,

particularly the metabolic burst of activated eosinophils, attracted and induced by activated microglia in the brain. While the overall experimental design and methodology are straightforward, there are concerns regarding the results and the presentation of findings in English.

**Ans: We thank the comment of reviewer and apologize for any errors that have appeared in the article. We have reviewed our English grammar and correct all the mistakes accordingly in the manuscript. (Throughout the text)**

**Reviewer #3:**

1. This study is the first to examine the brain of rats and mice infected by Angiostrongylus cantonensis using PET imaging, combined with cytokine profiling and histology and parasitological assessment. The overarching idea – is to get a better understanding of this disease in human patients by comparing the response seen in the permissive (definitive) host – the rat, with that in an accidental host – the mouse. Lots of elegant and complicated imaging and histology and cytokine profiling is done in these 2 rodent animal models.

But there are some inherent weaknesses in the study, The authors look at the brain, but not at the spinal cord, and some of the most severe pathological processes occur in the spinal cord.

**Ans: Thank you for your comment. In this study, we focused on applying PET imaging technology to analyze the inflammatory responses in the brains of animals of different species infected with *A. cantonensis*, especially emphasizing on immune differences in different brain regions. However, currently available PET animal image analytic modules are still mainly focused in mouse and rat brain templates. In addition, because *A. cantonensis* is still mainly located in the brain tissue for 2 to 4 weeks after infection in mice and rat, we first conducted the PET experiments that focused on the differences in the distribution of inflammation in the brain between these two species and its causes. In subsequent studies, we will try to expand the PET whole-body angiography to include the spinal cord region to observe the pathological effects of *A. cantonensis* infection in all CNS.**

2. And 50 large is a very heavy inoculum for either species, but especially in the mouse.

**Ans:** **Thank you for your comment. In our previous study, we infected 50 larvae in mice and 100 larvae in rats, and the results showed that it was feasible. These two studies have been added to the Materials section as references 38 and 39. Other studies have also shown that host infection with 50 L3 is a common method for ICR mice and does not result in severe mortality. Here we also provide two articles for reference. (Page8 line 145-146, ref. 38, 39)[****7-10****]**

3. If I had been doing these experiments, i think i would have used the guinea pig instead of the mouse – so that the permissive and non-permissive hosts were roughly the same side. And I would have done detailed neurological assessment of all infected animals, and undertaken PET imaging of the spinal cord as well as the brain and done histological assessment and cytokine profiling in the cord as well as different areas in the brain.

**Ans: Thanks to the reviewers for their suggestions. However, in previous studies, rats and mice were mostly and usually used as baselines for comparison because they both belong to the subfamily Murinae. Guinea pigs belong to the genus Cavia, which may make them significantly different from rats and mice in experimental settings. In addition, currently available PET animal image analysis modules are still mainly focused on mouse and rat brain templates, and there are no modules suitable for guinea pigs. We first used PET technology to detect the inflammatory responses in the brains of animals infected with *A. cantonensis.* We hope that the publication of this article will contribute to future detailed neurological assessments of infected hosts, including PET imaging, cytokine assessment and histology of different regions of the spinal cord and brain.**

4. And as the authors no doubt have access to excellent imaging, I would have been interested in high field MRI scans at the same time as the PET scanning.I am not convinced the patterns of inflammation seen in the mice would be all that different from a model of cryptococcosis, or a model of bacterial meningoencephalitis – so I think the notion that this will help diagnose human cases of rat lungworm disease is a bit of a stretch. And with the new Arcan qPCR that has been developed, combined with the new antigen detecting systems, the diagnosis of neural angiostrongyliasis is now both extremely sensitive and highly specific, so I really doubt PET will have a lot to add. Having said that – it is a shame the authors did not include a study from an affected human and dog with Angiostrongylus infection, as it might be that the spatial distribution of 8F/FDG/PET signal might be more characteristic in a MUCH LARGER brain and spinal cord.

**Ans: Thanks to the reviewers for their comments. We aim to develop a PET imaging platform that can be applied to analyze the inflammatory histopathology in different brain regions of animals infected with *A. cantonensis*. It is expected that it can be used for the treatment and prognosis assessment of brain lesions in angiostrongyliasis, rather than primarily for diagnosis. Therefore, we focus on the localization and interpretation of inflamed areas, which is more difficult to achieve with MRI, so we use PET with F18-FDG mode and hope that it can be used for other brain inflammatory diseases in the future. Secondly, our research is the first time to use PET imaging technology to analyze the brain symptoms of animal angiostrongyliasis. It is expected that after the evaluation of rats and mice is completed, its practical application will be further evaluated with inbred mice. In the future, preclinical dog experiments and clinical human trials are expected to be completed step by step. We also revised the related description in text. (Page4 line 71; page7 line130-131)**

5. The authors also need to revise the grammatical structure of the manuscript – as their tenses get all screwed up in the Method where they use the future tense.

**Ans: We thank the comment of reviewer and apologize for any errors that have appeared in the article. We have reviewed our English grammar and correct all the mistakes accordingly in the manuscript. (Throughout the text)**

6. I cannot really assess the histological sections as they are reproduced small, and do not have much spatial recognition.

**Ans: We apologize for the unclear histology part of the article. To make it easier to identify, we replaced the figures with a higher resolution in the manuscript. (Fig.2; Fig.3)**

1.	Kreutzberg GW. Microglia: a sensor for pathological events in the CNS. Trends Neurosci. 1996;19(8):312-8. Epub 1996/08/01. doi: 10.1016/0166-2236(96)10049-7. PubMed PMID: 8843599.

2.	Wu F, Wei J, Liu Z, Zeng X, Yu Z, Lv Z, et al. Soluble antigen derived from IV larva of Angiostrongylus cantonensis promotes chitinase-like protein 3 (Chil3) expression induced by interleukin-13. Parasitol Res. 2016;115(10):3737-46. Epub 2016/06/04. doi: 10.1007/s00436-016-5135-x. PubMed PMID: 27256220.

3.	Zhao J, Lv Z, Wang F, Wei J, Zhang Q, Li S, et al. Ym1, an eosinophilic chemotactic factor, participates in the brain inflammation induced by Angiostrongylus cantonensis in mice. Parasitol Res. 2013;112(7):2689-95. Epub 2013/05/25. doi: 10.1007/s00436-013-3436-x. PubMed PMID: 23703548.

4.	Nimmerjahn A, Kirchhoff F, Helmchen F. Resting microglial cells are highly dynamic surveillants of brain parenchyma in vivo. Science. 2005;308(5726):1314-8. Epub 2005/04/16. doi: 10.1126/science.1110647. PubMed PMID: 15831717.

5.	Tran GT, Wilcox PL, Dent LA, Robinson CM, Carter N, Verma ND, et al. Interleukin-5 Mediates Parasite-Induced Protection against Experimental Autoimmune Encephalomyelitis: Association with Induction of Antigen-Specific CD4(+)CD25(+) T Regulatory Cells. Front Immunol. 2017;8:1453. Epub 2017/11/23. doi: 10.3389/fimmu.2017.01453. PubMed PMID: 29163523; PubMed Central PMCID: PMCPMC5671975.

6.	Greenfeder S, Umland SP, Cuss FM, Chapman RW, Egan RW. Th2 cytokines and asthma. The role of interleukin-5 in allergic eosinophilic disease. Respir Res. 2001;2(2):71-9. Epub 2001/11/01. doi: 10.1186/rr41. PubMed PMID: 11686868; PubMed Central PMCID: PMCPMC59571.

7.	Lee CY, Liao CW, Wang LC, Fan CK, Chuang TW, En-Te Hwu E, et al. IgE antibody responses in cerebrospinal fluids relate to the brain pathologic injury of hosts with Angiostrongylus cantonensis infection. J Microbiol Immunol Infect. 2023. Epub 2023/09/10. doi: 10.1016/j.jmii.2023.08.012. PubMed PMID: 37689501.

8.	Lan KP, Lai SC. Differences of proteolytic enzymes and pathological changes in permissive and nonpermissive animal hosts for Angiostrongylus cantonensis infection. Vet Parasitol. 2009;165(3-4):265-72. Epub 2009/08/13. doi: 10.1016/j.vetpar.2009.07.029. PubMed PMID: 19671486.

9.	Tsai HC, Chen YH, Yen CM, Lee SS, Chen YS. Increased 14-3-3beta and gamma protein isoform expressions in parasitic eosinophilic meningitis caused by Angiostrongylus cantonensis infection in mice. PLoS One. 2019;14(3):e0213244. Epub 2019/03/08. doi: 10.1371/journal.pone.0213244. PubMed PMID: 30845271; PubMed Central PMCID: PMCPMC6405114.

10.	Lee CY, Cheng PC, Ho CH, Fan CK, Chao D. Immunologic evaluation of extracted intestinal proteins from Angiostrongylus cantonensis adult worms. J Microbiol Immunol Infect. 2019;52(4):620-9. Epub 2017/08/06. doi: 10.1016/j.jmii.2017.07.008. PubMed PMID: 28778593.
